# Supplementary material for: Motivating factors, barriers and facilitators of participation in COVID-19 clinical research: A cross-sectional survey of Canadian community intensive care units
Source: PLoS One. 2022 Apr 27;17(4):e0266770. doi: 10.1371/journal.pone.0266770 (PMC9045667; doi:10.1371/journal.pone.0266770)
Supplement: S1 Appendix — (PDF) [file pone.0266770.s002.pdf]

Covid-Community ICU Survey

2020-06-01 v6

A. DEMOGRAPHICS

Please answer the following questions with respect to the COMMUNITY ICU at which you work the most.

If you work in BOTH a community ICU and an academic ICU, PLEASE DESCRIBE THE COMMUNITY ICU. If you work in a commnity hospital centre with more than one campus, PLEASE DESCRIBE THE ICU AT WHICH YOU WORK THE MOST.

date completed

____

1. My hospital name is:

Note: The name of your hospital is requested for the purposes of combining responses from the same institution. No hospital names will be reported in the study and data from multiple hospitals will be aggregated to ensure anonymity of all institutions.

____

2. My hospital is located in (town/city):

____

3. My Hospital is located in (province)

◯ Prince Edward Island ◯ Newfoundland ◯ Nova Scotia ◯ New Brunswick ◯ Quebec
◯ Ontario ◯ Saskatchewan ◯ Manitoba ◯ Alberta ◯ British Columbia ◯ North West Territories
◯ Yukon ◯ Nunavut

4. My primary role in the ICU is:
(Select 1 response only)

◯ Physician
◯ Nurse
◯ Pharmacist
◯ Respiratory Therapist
◯ Physiotherapist
◯ Occupational Therapist
◯ Dietician
◯ Research Coordinator/Research Assistant
◯ Research Management/Administration or Hospital Administrator
◯ Other

Specify primary role

____

4a. My gender is:

◯ Female
◯ Male
◯ Non-binary
◯ Other
◯ Prefer not to say

Specify other gender

____

4b. I have been practicing in the ICU for:

◯ < 1 year
◯ 1 to 5 years
◯ 6 to 10 years
◯ 11 to 20 years
◯ > 20 years

4c. What previous research experience have you had PERSONALLY? (check all that apply):

☐ Clinical research as a principle investigator
☐ Clinical research as a local site investigator or co-investigator
☐ Clinical research as a research coordinator or research assistant
☐ Caring for patients enrolled in clinical research studies
☐ Research training at the graduate level (e.g. Master's degree, PhD degree)
☐ Research experience as a trainee (student, resident)
☐ Online research courses (e.g. Good Clinical Practice, Division 5 training, etc)
☐ Basic science research experience (i.e. laboratory research)
☐ Other

List other previous research experiences:

5. The ICU that I work in is:

◯ Closed (patients are cared for principally by the intensive care physicians)
◯ Open (patients are cared for principally by a non-intensive care physician with support from an intensivist or anesthetist)
◯ Both closed and open
◯ Other

Describe the model of ICU in which you work

____

6. The patient population in the ICU that I predominantly work in is best characterized as (check all that apply):

- ☐ Medical
- ☐ Surgical
- ☐ Trauma
- ☐ Burns
- ☐ Cardiovascular surgical
- ☐ Coronary Care
- ☐ Neurosurgical
- ☐ Transplant
- ☐ Other

Specify the patient population in the ICU

____

7. Please indicate the number of ICU beds in the ICU that you work in under normal conditions (i.e., pre-COVID-19 pandemic)

Level 3 (ventilated)

◯ 0
◯ 1
◯ 2
◯ 3
◯ 4
◯ 5
◯ 6
◯ 7
◯ 8
◯ 9
◯ 10
◯ 11
◯ 12
◯ 13
◯ 14
◯ 15
◯ 16
◯ 17
◯ 18
◯ 19
◯ 20
◯ 21
◯ 22
◯ 23
◯ 24
◯ 25
◯ 26
◯ 27
◯ 28
◯ 29
◯ 30
◯ 31
◯ 32
◯ 33
◯ 34
◯ 35
◯ 36
◯ 37
◯ 38
◯ 39
◯ 40
◯ 41
◯ 42
◯ 43
◯ 44
◯ 45
◯ 46
◯ 47
◯ 48
◯ 49
◯ 50

Level 2 (non-ventilated, step-down)

◯ 0
◯ 1
◯ 2
◯ 3
◯ 4
◯ 5
◯ 6
◯ 7
◯ 8
◯ 9
◯ 10
◯ 11
◯ 12
◯ 13
◯ 14
◯ 15
◯ 16
◯ 17
◯ 18
◯ 19
◯ 20
◯ 21
◯ 22
◯ 23
◯ 24
◯ 25
◯ 26
◯ 27
◯ 28
◯ 29
◯ 30
◯ 31
◯ 32
◯ 33
◯ 34
◯ 35
◯ 36
◯ 37
◯ 38
◯ 39
◯ 40
◯ 41
◯ 42
◯ 43
◯ 44
◯ 45
◯ 46
◯ 47
◯ 48
◯ 49
◯ 50

8. Please indicate the number of ICU beds in the ICU that you work in during COVID-19 pandemic conditions

Level 3 (ventilated)

◯ 0
◯ 1
◯ 2
◯ 3
◯ 4
◯ 5
◯ 6
◯ 7
◯ 8
◯ 9
◯ 10
◯ 11
◯ 12
◯ 13
◯ 14
◯ 15
◯ 16
◯ 17
◯ 18
◯ 19
◯ 20
◯ 21
◯ 22
◯ 23
◯ 24
◯ 25
◯ 26
◯ 27
◯ 28
◯ 29
◯ 30
◯ 31
◯ 32
◯ 33
◯ 34
◯ 35
◯ 36
◯ 37
◯ 38
◯ 39
◯ 40
◯ 41
◯ 42
◯ 43
◯ 44
◯ 45
◯ 46
◯ 47
◯ 48
◯ 49
◯ 50

Level 2 (non-ventilated, step-down)

◯ 0
◯ 1
◯ 2
◯ 3
◯ 4
◯ 5
◯ 6
◯ 7
◯ 8
◯ 9
◯ 10
◯ 11
◯ 12
◯ 13
◯ 14
◯ 15
◯ 16
◯ 17
◯ 18
◯ 19
◯ 20
◯ 21
◯ 22
◯ 23
◯ 24
◯ 25
◯ 26
◯ 27
◯ 28
◯ 29
◯ 30
◯ 31
◯ 32
◯ 33
◯ 34
◯ 35
◯ 36
◯ 37
◯ 38
◯ 39
◯ 40
◯ 41
◯ 42
◯ 43
◯ 44
◯ 45
◯ 46
◯ 47
◯ 48
◯ 49
◯ 50

COVID-19 Patients

9a. At the peak of the COVID-19 pandemic to date, what was the greatest number of COVID-19 patients cared for in your ICU at one time?

◯ 0
◯ 1 to 5
◯ 6 to10
◯ 11 to 15
◯ 16 to 20
◯ 21 to 30
◯ more than 30

9b. Record the month in 2020 when you had the highest number of COVID-19 patients in your ICU

◯ Unknown
◯ February
◯ March
◯ April
◯ May
◯ June
◯ July
◯ August
◯ September
◯ October
◯ November
◯ December

B. RESOURCE FOR RESERACH

10. Do you have an electronic medical record (EMR) in your ICU?

◯ Yes
◯ No

What is the name of your EMR?

____

10a. Which of the following capabilities does your EMR have? (check all that apply)

☐ Patient reports / results viewing
☐ Patient vitals / monitoring entry and viewing
☐ Daily healthcare professional notes
☐ Order entry
☐ Display imaging studies
☐ Ramote access (e.g., accessible from home)
☐ Daily healthcare professional notes
☐ Entry
☐ Viewing
☐ Other

Specify other capabilities your EMR has

11. What pre-existing research infrastructure do you have available at your ICU/hospital (check all that apply):

☐ Pre-existing ICU research program
☐ Pre-existing research program(s) in the hospital (other departments)
☐ Research coordinator(s) or research assistant(s) in the ICU
☐ Research coordinator(s) or research assistant(s) in other department(s) in the hospital
☐ Local research ethics board
☐ Remote research ethics board (i.e. REB at another hospital)
☐ Hospital research administration/office
☐ On-site contract review capability
☐ Research policies and procedures
☐ Pharmacy department with research capability or experience
☐ Clinical laboratory department with research capability or experience
☐ Diagnostic imaging department with research capability or experience
☐ Other

Specify other pre-existing research infrastructure you have available

11a. Which research programs exist in your hospital? (check all that apply):

☐ Oncology
☐ Cardiology
☐ Nephrology
☐ ICU
☐ Other (specify below)

Specify other research programs in your hospital

____

12. If your ICU has participated or is currently participating in clinical research, please list the names of the current studies and those that have been conducted in your ICU in the last 5 years.

13. Please rate the degree of importance of the following infrastructures in facilitating the participation of your ICU in clinical research studies.

		Not important	1	2	3	4	5 Very Important
a	Dedicated external funding to provide start-up costs for a research program	○	○	○	○	○	○
b	Dedicated external funding to sustain ongoing costs for a research program	○	○	○	○	○	○
c	The existence of a more positive "research culture" in my hospital	○	○	○	○	○	○
d	The existence of a more positive "research culture" amongst ICU staff and physicians	○	○	○	○	○	○
e	Administrative support for research infrastructure from my hospital (e.g., contract review, research ethics board, policies and procedures, research office)	○	○	○	○	○	○
f	Pharmacy department with capacity (e.g., personnel) to randomize and dispense study drugs	○	○	○	○	○	○
g	Availability of experienced research coordinators	○	○	○	○	○	○
h	Research mentorship within my hospital	○	○	○	○	○	○
i	Partnership with academic hospitals	○	○	○	○	○	○
j	Participation in research networks that support research in community hospitals	○	○	○	○	○	○

Please list any other factor(s) that would FACILITATE YOUR participation in pandemic-related ICU research.

C. MOTIVATING FACTORS TO PARTICIPATE IN COVID-19 RESERACH

14. Please rate your degree of interest in participating in COVID-19 pandemic research

◯ 1=Very uninterested
◯ 2
◯ 3
◯ 4
◯ 5
◯ 6
◯ 7 = Very interested

15. Please explain why you are interested or not interested

16. The following MOTIVATING FACTORS are important for your participation in research. Please indicate the level of your disagreement or agreement with the following.

		Strongly disagree	Disagree	Neither agree or disagree	Agree	Strongly agree
a.	Improve care and clinical outcomes	◯	◯	◯	◯	◯
b.	Advance medical knowledge	◯	◯	◯	◯	◯
c.	Increase the recognition and credibility of my hospital	◯	◯	◯	◯	◯
d.	Enhance professional opportunities in the hospital	◯	◯	◯	◯	◯
e.	Increase staff retention	◯	◯	◯	◯	◯
f.	Increase staff career satisfaction	◯	◯	◯	◯	◯
g.	Help attract healthcare professionals to hospital	◯	◯	◯	◯	◯
h.	Establish collaborations with other ICUs/ICU research	◯	◯	◯	◯	◯
i.	Stay informed about current reserach	◯	◯	◯	◯	◯
j.	Provide opportunities to attend conferences	◯	◯	◯	◯	◯
k.	Provide opportunities for work related travel	◯	◯	◯	◯	◯
l.	Enhance, diversity or advance my career	◯	◯	◯	◯	◯

17. Please list any other MOTIVATING FACTOR(S) that increase YOUR interest in participating in community ICU research

D. BARRIERS TO PARTICIPATION IN COMMUNITY ICU RESEARCH

18. The following items are significant BARRIERS to your participation in research. Please indicate the level of your disagreement or agreement with the following.

		Strongly disagree	Disagree	Neither agree or disagree	Agree	Strongly agree
a.	Community ICUs ar not known or expected to do reserach (identity)	○	○	○	○	○
b.	Lack of interest on the part of the ICU staff and physicians in my unit (culture)	○	○	○	○	○
c.	Lack of reserach experience in our ICU	○	○	○	○	○
d.	Lack of start-up funding for a reserach coordinator	○	○	○	○	○
e.	Inadequate per-patient payments from study sponsors to sustain research coordinator salary	○	○	○	○	○
f.	Staff workload is too high due to pandemic-related pressures	○	○	○	○	○
g.	Physicians not willing to dedicate unpaid time to reserach	○	○	○	○	○
h.	Negative experience(s) with research in the past	○	○	○	○	○
i.	Hospital moratorium on research during pandemic	○	○	○	○	○
j.	Fear related to COVID-19 and/or availability of PPE in ICU	○	○	○	○	○

Please list any other BARRIER(S) to YOUR participation in community ICU research

E. QUESTIONS ON POTENTIAL RESERACH PROGRAM MODELS

19. There may be funding available to assist Canadian community ICUs with participating in pandemic-related research. Several models have been proposed to assist Canadian community ICUs with participating in pandemic-related research should funding be available. Please review the following models.

Model A:
• Study sponsor will subsidize a portion of research coordinator salary (i.e. 30-50%) for 6-12 months to enable ICU to start-up a local research program
• Local site will provide an MD to act as site investigator who will help the RC to get the study up and running locally
• ICU/hospital will hire research coordinator locally
• ICU will be free to enrol patients for multiple studies and can continue program after sponsorship period finishes

Model B:
• Sponsor will hire and pay a research coordinator who will recruit patients for a single research study
• Local site will provide an MD to act as site investigator who will help the RC to get the study up and running locally
• The research coordinator will work remotely (i.e. will obtain consent over the phone, access EMR remotely, coordinate study enrolment with MDs and RNs by phone)
• The RC will NOT be available to work on other studies and the arrangement will terminate at the end of the study

Model C:
• Sponsor will hire and pay a research coordinator who will recruit patients for a package of studies (pandemic and non-pandemic)
• Local site will provide a MDs to act as site investigators and will help the RC to get the studies up and running locally
• The research coordinator will work on-site to coordinate the studies
• The per-patient payments will revert to the sponsors, not to the hospital/ICU
• After the end of the sponsorship period, the hospital/ICU will be free to decide if they wish to continue research and to hire a research coordinator locally

After reviewing the Models above I would prefer:

◯ Model A
◯ Model B
◯ Model C

---

20. Please explain your choice of Model

---

21. Is there an alternative model that you think would work better? If so, please indicate below.
