# Supplementary material for: Motivating factors, barriers and facilitators of participation in COVID-19 clinical research: A cross-sectional survey of Canadian community intensive care units
Source: PLoS One. 2022 Apr 27;17(4):e0266770. doi: 10.1371/journal.pone.0266770 (PMC9045667; doi:10.1371/journal.pone.0266770)
Supplement: Attachment — Submitted filename: Response to Reviewers Comments 2022-03-06.docx [file pone.0266770.s003.docx]

Dear PLOS One Editors and Reviewers,

Thank you for spending the time reviewing our manuscript despite your busy schedule and providing comments.

We have made changes to our manuscript as suggested by editors and reviewers. Please see our responses in red below.

We have also made some minor editorial changes to the rest of the manuscript. These changes are marked via tracked changes. I have included both the tracked changes file and clean file.

Reviewer #1: For abstract: Include mean/SD/p value in results section Mean and SD included. P value is not applicable here.

Conclusion for abstract I am unclear on what is requested here.

Cite line 73 (introduction) Completed

Thank you for the comment. We have made the above changes accordingly.

The study was well conducted and written.
Hope it will give the merits of improving COVID 19 research in your community and elsewhere.

Thank you.

Reviewer #2: This is an online survey, and as such it gives useful information about what Canadian intensivists think is needed to increase clinical research. Most of the critical factors identified as motivating, facilitating or obstructing, are probably of general validity.

Thank you.

1. Please ensure that your manuscript meets PLOS ONE's style requirements, including those for file naming. Completed. The PLOS ONE style templates can be found at

https://journals.plos.org/plosone/s/file?id=wjVg/PLOSOne_formatting_sample_main_body.pdf and https://journals.plos.org/plosone/s/file?id=wjVg/PLOSOne_formatting_sample_main_body.pdf and https://journals.plos.org/plosone/s/file?id=ba62/PLOSOne_formatting_sample_title_authors_affiliations.pdf

Please review your reference list to ensure that it is complete and correct. If you have cited papers that have been retracted, please include the rationale for doing so in the manuscript text, or remove these references and replace them with relevant current references. Any changes to the reference list should be mentioned in the rebuttal letter that accompanies your revised manuscript. If you need to cite a retracted article, indicate the article’s retracted status in the References list and also include a citation and full reference for the retraction notice.

Done.

2. Please provide additional details regarding participant consent. In the ethics statement in the Methods and online submission information, please ensure that you have specified (1) whether consent was informed and (2) what type you obtained (for instance, written or verbal, and if verbal, how it was documented and witnessed). If your study included minors, state whether you obtained consent from parents or guardians. If the need for consent was waived by the ethics committee, please include this information. Informed consent was obtained electronically via online survey tool (REDCap). We have amended the manuscript in ethics statement and online submission information.

If you are reporting a retrospective study of medical records or archived samples, please ensure that you have discussed whether all data were fully anonymized before you accessed them and/or whether the IRB or ethics committee waived the requirement for informed consent. If patients provided informed written consent to have data from their medical records used in research, please include this information. N/A

3. We note that you have stated that you will provide repository information for your data at acceptance. Should your manuscript be accepted for publication, we will hold it until you provide the relevant accession numbers or DOIs necessary to access your data. If you wish to make changes to your Data Availability statement, please describe these changes in your cover letter and we will update your Data Availability statement to reflect the information you provide Will do.

Thank you. We look forward to hearing from you.

Jennifer Tsang, MD, PhD, FRCPC, ABOM Diplomate

Research Lead, Intensivist, Niagara Health

Physician Co-Lead, Critical Care Research Program, Niagara Health

Regional Deputy Research Director, Internal Medicine Residency Program, McMaster University

Associate Professor of Medicine, McMaster University

March 6, 2022
